# A Robust and Effective Smart-Card-Based Remote User Authentication Mechanism Using Hash Function

**DOI:** 10.1155/2014/719470

**Published:** 2014-04-29

**Authors:** Ashok Kumar Das, Vanga Odelu, Adrijit Goswami

**Affiliations:** ^1^Center for Security, Theory and Algorithmic Research, International Institute of Information Technology, Hyderabad 500 032, India; ^2^Department of Mathematics, Indian Institute of Technology, Kharagpur 721 302, India

## Abstract

In a remote user authentication scheme, a remote server verifies whether a login user is genuine and trustworthy, and also for mutual authentication purpose a login user validates whether the remote server is genuine and trustworthy. Several remote user authentication schemes using the password, the biometrics, and the smart card have been proposed in the literature. However, most schemes proposed in the literature are either computationally expensive or insecure against several known attacks. In this paper, we aim to propose a new robust and effective password-based remote user authentication scheme using smart card. Our scheme is efficient, because our scheme uses only efficient one-way hash function and bitwise XOR operations. Through the rigorous informal and formal security analysis, we show that our scheme is secure against possible known attacks. We perform the simulation for the formal security analysis using the widely accepted AVISPA (Automated Validation Internet Security Protocols and Applications) tool to ensure that our scheme is secure against passive and active attacks. Furthermore, our scheme supports efficiently the password change phase always locally without contacting the remote server and correctly. In addition, our scheme performs significantly better than other existing schemes in terms of communication, computational overheads, security, and features provided by our scheme.

## 1. Introduction

In recent years, the remote user authentication using smart cards has become an important research area in computer science. In remote user authentication, communicating parties are verified as to whether they are genuine and trustworthy and the users are authenticated by a remote server before allowing access to services. Several password-based schemes (e.g., [1–3]) or biometric-based schemes (e.g., [4–6]) have been proposed for remote user authentication problem. An idle password-based remote user authentication scheme using smart cards needs to satisfy the following requirements [2]:

- not maintaining verification tables;
- user's ability to freely choose and update password;
- resistance to password disclosure to the server;
- prevention of masquerade attacks;
- resistance to replay, modification, parallel session, and stolen-verifier attacks;
- an easy-to-remember password;
- low communication cost and computation complexity;
- achieving mutual authentication between login users and remote servers;
- resistance to guessing attacks even if the smart card is lost or stolen by attackers;
- session key agreement;
- resistance to insider attacks;
- prevention of smart card security breach attacks.

The majority of the proposed password-based remote user authentication schemes are either computationally expensive or vulnerable to different known attacks. Some comprehensive surveys on password-based remote user authentication schemes could be found in [7, 8]. Das et al. [9] proposed a dynamic ID and password-based remote user authentication scheme using smart cards, which uses the efficient hash function and bitwise XOR operations. However, Wang et al. [10] showed that Das et al.'s scheme is vulnerable to different attacks and it does not achieve mutual authentication property and does not resist impersonating remote server attack. Wang et al. then proposed an enhancement of their scheme using smart cards. Later, Khan et al. [11] analyzed the security of Wang et al.'s scheme and they showed that Wang et al.'s scheme has several weaknesses, for example, it does not provide anonymity of a user during authentication, the user has no choice in choosing his/her password, it is vulnerable to insider attack, it has no provision for revocation of lost or stolen smart card, and, finally, it does not provide session key agreement. In order to remedy these security weaknesses, Khan et al. also proposed an enhanced password-based remote user authentication scheme using smart cards.

In 2012, Sonwanshi et al. [3] proposed a password-based remote user authentication scheme using the smart card, which uses only the one-way hash function and bitwise XOR operation. However, in 2013, Das et al. [12] showed that their scheme is vulnerable to the offline password guessing attack and stolen smart card attack. In addition, Das et al. showed that their scheme fails to protect strong replay attack. In 2013, Lee and Liu [13] proposed a password-based authentication and key agreement scheme, which uses the public-key cryptosystem and one-way hash function. Lee-Liu's scheme is expensive in computation as it requires expensive modular exponentiation operations. Lee-Liu's scheme supports session key security and protects against parallel session attack, password guessing attack, privileged insider attack, replay attack, and man-in-the-middle attack. Their scheme also provides user's anonymity property. In 2013, Das and Bruhadeshwar [14] showed that Lee-Liu's scheme has two security weaknesses: (1) it has design flaws in authentication phase and (2) it has design flaws in password change phase. In order to withstand these flaws found in Lee-Liu's scheme, they proposed an improved and effective password-based remote user authentication scheme. However, Das-Bruhadeshwar's scheme [14] is also computationally costly as it requires expensive modular exponentiation operations. Recently, in 2013, Jiang et al. [15] proposed a secure password-based remote user authentication scheme without pairings for multiserver architecture. However, their scheme uses ECC (elliptic curve cryptography) cryptosystem and hash function. Due to expensive ECC point addition and scalar multiplication operations, their scheme is also expensive.

In this paper, we propose a new robust and secure password-based remote user authentication scheme using the one-way hash function and bitwise XOR operation only. The rest of this paper is organized as follows. In Section 2, we give a mathematical background on the one-way hash function, which will be helpful for describing and analyzing our scheme. In Section 3, we propose our new robust and secure password-based remote user authentication scheme. In Section 4, we analyze our scheme under different possible attacks using both the informal and formal security analysis. In Section 5, we perform the simulation for the formal security analysis using the widely accepted AVISPA (Automated Validation of Internet Security Protocols and Applications) tool to ensure that our scheme is secure against passive and active attacks. In Section 6, we compare the performance of our scheme with the recently proposed password-based remote user authentication schemes [3, 13–15]. Finally, we conclude the paper in Section 7.

## 2. Mathematical Preliminaries

In this section, we discuss the properties of one-way hash function for describing and analyzing our scheme.

A hash function *h* : {0,1}* → {0,1}^*n*^ is a one-way function, which takes an arbitrary-length binary string input *x* ∈ {0,1}* and outputs a fixed-length (e.g., *n*-bit) binary string, called the message digest or hash value *h*(*x*)∈{0,1}^*n*^. In addition, it has the following important properties [16].

- *h* can be applied to a data block of all sizes.
- For any given input *x*, it is relatively easy to compute the hash value *h*(*x*), which enables easy implementation in software and hardware.
- Output length of *h*(*x*) is fixed.
- One-way property: from a given hash value *y* = *h*(*x*) and the given hash function *h*(·), it is computationally infeasible to derive the input *x*.
- Weak-collision resistance property: for any given input *x*, finding any other input *y*, with *y* ≠ *x*, such that *h*(*y*) = *h*(*x*) is computationally infeasible.
- Strong-collision resistance property: finding a pair of inputs (*x*, *y*), with *x* ≠ *y*, such that *h*(*x*) = *h*(*y*) is also computationally infeasible.

An example of such a one-way function is SHA-1 [17], which has the above desired properties. At present, the National Institute of Standards and Technology (NIST) does not recommend SHA-1 for top secret documents. In 2011, Manuel [18] showed the collision attacks on SHA-1. Quark [19] is a family of cryptographic hash functions, which is designed for extremely resource-constrained environments like sensor networks and radiofrequency identification tags. Like most one-way hash functions, Quark can be used as a pseudorandom function, a message authentication code, a pseudorandom number generator, a key derivation function, and so forth. Quark performs better than the SHA-1 hash function. Thus, Quark can be used for the one-way function. However, in this paper, as in [14, 20, 21], we can use SHA-2 as the secure one-way hash function in order to achieve top security, whereas we use only 160 bits from the hash digest output of SHA-2 in our scheme and other schemes.

## 3. The Proposed Scheme

In this section, we propose a new remote user authentication scheme using password, which is based on smart card. For this purpose, we first discuss the threat model used in our scheme. We then discuss the various phases related to our scheme.

### 3.1. Notations

For describing and analyzing our scheme, we use the notations listed as follows:

 
*U*_*i*_: user,
 
*S*_*j*_: remote server,
 
*ID*_*i*_: identity of user *U*_*i*_,
 
*PW*_*i*_: password of user *U*_*i*_,
 
*X*_*s*_: permanent secret key only known to the remote server *S*_*j*_,
 
*K*: secret number only known to the user *U*_*i*_,
 
*T*_*a*_: current system timestamp of an entity *A*,
 
*R*_*a*_: random nonce generated by an entity *A*,
 
*h*(·): secure one-way collision-resistant hash function,
 
*A*||*B*: data *A* concatenating with data *B*,
 
*A* ⊕ *B*: bitwise XOR operation of *A* and *B*.

### 3.2. Threat Model

In our scheme, we make use of the Dolev-Yao threat model [22]. In this model, two communicating parties communicate over an insecure channel. Any adversary (attacker or intruder) can thus eavesdrop on the transmitted messages over the public insecure channel and he/she has the ability to modify, delete, or change the contents of the transmitted messages. Usually, the smart card issued to a user is equipped with tamper-resistant device. However, in this paper, we still assume that once a user's smart card is stolen or lost, the attacker will know all the sensitive information stored in the smart card's memory by monitoring the power consumption of the smart card [23, 24].

### 3.3. Motivation

The majority of the proposed password-based remote user authentication schemes are either computationally expensive or vulnerable to different known attacks [7, 8]. Though Sonwanshi et al.'s scheme [3] is very efficient due to usage of one-way hash function and bitwise XOR operations, Das et al. [12] showed that their scheme is vulnerable to the offline password guessing attack and stolen smart card attack. In addition, Das et al. showed that their scheme fails to protect strong replay attack. Lee-Liu's scheme [13] is expensive in computation as it requires expensive modular exponentiation operations. Further, Das and Bruhadeshwar [14] showed that Lee-Liu's scheme has security weaknesses. In order to withstand the flaws found in Lee-Liu's scheme, they proposed an improved and secure password-based remote user authentication scheme. However, Das-Bruhadeshwar's scheme [14] is also computationally costly as it requires expensive modular exponentiation operations as in Lee-Liu's scheme [13]. The recently proposed Jiang et al.'s scheme [15] uses ECC cryptosystem and hash function. Due to expensive ECC point addition and scalar multiplication operations, their scheme is also expensive, though their scheme is secure against different attacks. Thus, we feel that there is a great need to propose a new robust and secure password-based remote user authentication scheme which will satisfy the requirements listed in Section 1. Our scheme withstands the security flaws found in Sonwanshi et al.'s scheme [3] and it is also very efficient as our scheme relies only on lightweight operations like the one-way hash computations and bitwise XOR operations.

### 3.4. Different Phases

In this section, we describe the four phases related to our scheme, namely, the registration phase, the login phase, the authentication phase, and the password change phase. In the registration phase, a user *U*_*i*_ needs to register to access services from a remote server *S*_*j*_. After registering, the server *S*_*j*_ will issue a smart card containing important information stored in the smart card's memory. In the login phase, if the user *U*_*i*_ wants to access services from the server *S*_*j*_, the user *U*_*i*_ needs to login to the system providing his/her identity and password with the help of his/her smart card issued by the registration server. In the authentication phase, the server *S*_*j*_ authenticates the user *U*_*i*_ and the user *U*_*i*_ also authenticates the server *S*_*j*_. After mutual authentication between *U*_*i*_ and *S*_*j*_, both *U*_*i*_ and *S*_*j*_ establish a secret common session key shared between them so that they communicate securely using that established key in future.

#### 3.4.1. Registration Phase

This phase consists of the following steps.

*Step R1*. The user *U*_*i*_ first selects his/her own secret identity *ID*_*i*_ and chooses a strong (not low-entropy or weak) password *PW*_*i*_.

*Step R2*. *U*_*i*_ then generates a secret 1024-bit number *K* randomly, which is kept secret to *U*_*i*_ only.

*Step R3*. *U*_*i*_ then computes the masked password using *K*, *ID*_*i*_, and *PW*_*i*_ as *R* *PW*_*i*_ = *h*(*ID*_*i*_||*K*||*PW*_*i*_) and sends the registration request message 〈*ID*_*i*_, *R* *PW*_*i*_〉 to the registration remote server *S*_*j*_ via a secure channel.

*Step R4*. After receiving the registration request message in Step R3, the server *S*_*j*_ generates a 1024-bit secret number *X*_*s*_ randomly, which is kept secret to *S*_*j*_ only.

*Step R5*. *S*_*j*_ then computes *r*_*i*_ = *h*(*ID*_*i*_||*R* *PW*_*i*_) = *h*(*ID*_*i*_||*h*(*ID*_*i*_||*K*||*PW*_*i*_)) and *e*_*i*_ = *h*(*ID*_*i*_||*X*_*s*_) ⊕ *r*_*i*_. *S*_*j*_ further computes *TD*_*i*_ = *N* *ID*_*i*_ ⊕ *h*(*ID*_*i*_||*r*_*i*_) and *D*_*i*_ = *TD*_*i*_ as in [20]. Here *N* *ID*_*i*_ is a random and temporary identity for the user *U*_*i*_, which is used instead of the permanent identity *ID*_*i*_ to achieve the user anonymity.

*Step R6*. Finally, *S*_*j*_ issues a smart card *C*_*i*_ containing the information (*r*_*i*_, *e*_*i*_, *TD*_*i*_, *D*_*i*_, *h*(·)) and sends it to the user *U*_*i*_ via a secure channel.

After receiving the smart card *C*_*i*_ from *S*_*j*_, *U*_*i*_ stores the secret number *K* into the smart card's memory. The summary of the registration phase is given in Table 1.

#### 3.4.2. Login Phase

In this phase, the following steps are executed.

*Step L1*. *U*_*i*_ first inserts his/her smart card *C*_*i*_ into a card reader of the specific terminal. *U*_*i*_ then inputs his/her identity *ID*_i_* and password *PW*_i_*.

*Step L2*. *C*_*i*_ computes the masked password *R* *PW*_i_* as *R* *PW*_i_* = *h*(*ID*_i_*||*K*||*PW*_i_*) using the secret number *K* stored in its memory. *C*_*i*_ then computes *r*_i_* = *h*(*ID*_i_*||*R* *PW*_i_*) and checks if the condition *r*_i_* = *r*_*i*_ holds. If this condition holds, *U*_*i*_ passes password verification and the next step is executed. Otherwise, this phase terminates immediately.

*Step L3*. *C*_*i*_ computes *N* *ID*_i_* = *h*(*ID*_i_*||*r*_i_*) ⊕ *D*_*i*_ and *M*_1_ = *e*_*i*_ ⊕ *r*_i_* = *h*(*ID*_*i*_||*X*_*s*_) ⊕ *r*_*i*_ ⊕ *r*_i_* = *h*(*ID*_*i*_||*X*_*s*_). *C*_*i*_ generates a 160-bit random nonce *R*_*c*_ and then computes *M*_2_ = *M*_1_ ⊕ *R*_*c*_ ⊕ *T*_*c*_ = *h*(*ID*_*i*_||*X*_*s*_) ⊕ *R*_*c*_ ⊕ *T*_*c*_, where *T*_*c*_ is the current system timestamp, and *M*_3_ = *h*(*ID*_i_*||*R*_*c*_||*T*_*c*_). *C*_*i*_ sends the login request message 〈*N* *ID*_i_*, *M*_2_, *M*_3_, *T*_*c*_〉 to the server *S*_*j*_ via a public channel.

The summary of the login phase is given in Table 2.

#### 3.4.3. Authentication Phase

After receiving the login request message 〈*N* *ID*_i_*, *M*_2_, *M*_3_, *T*_*c*_〉 from the user *U*_*i*_, the server *S*_*j*_ checks the format of *N* *ID*_i_* and then finds the entry (*ID*_*i*_, *N* *ID*_i_*) in its maintained ID database table. If it is found, *S*_*j*_ performs Case 1; otherwise, *S*_*j*_ proceeds to Case 2.

**Case 1**

Consider the following.

*Step A1*. *S*_*j*_ checks the validity of the timestamp *T*_*c*_ in the received message by the condition |*T*_*c*_ − *T*_c_* | <Δ*T*, where *T*_c_* is the current system timestamp of *S*_*j*_ and Δ*T* the expected transmission delay. If this condition is satisfied, *S*_*j*_ computes *M*_4_ = *h*(*ID*_*i*_||*X*_*s*_), using its own secret number *X*_*s*_. After that *S*_*j*_ computes

(1) M5=M2⊕M4⊕Tc   =h(IDi||Xs)⊕Rc⊕Tc⊕h(IDi||Xs)⊕Tc   =Rc,M6=h(IDi||M5||Tc).

*S*_*j*_ then verifies the condition whether *M*_6_ = *M*_3_ holds. If it does not hold, *S*_*j*_ rejects the login request message and this phase terminates immediately.

In order to protect the man-in-the-middle attacks and the replay attacks, we can adopt the same strategy as in [4, 20]. The server *S*_*j*_ stores the pair (*ID*_*i*_, *M*_5_), where *M*_5_ = *R*_*c*_, in its database. Suppose the server receives the next login request message 〈*N* *ID*_i_*, *M*_2_′, *M*_3_′, *T*_*c*_′〉 from the user *U*_*i*_ or an attacker. *S*_*j*_ first checks the validity of the timestamp *T*_*c*_′ and if it is valid, it further computes *M*_4_′ = *h*(*ID*_*i*_||*X*_*s*_), using its own secret number *X*_*s*_. After that *S*_*j*_ computes, say, *M*_5_′ = *M*_2_′ ⊕ *M*_4_′ ⊕ *T*_*c*_′ = *h*(*ID*_*i*_||*X*_*s*_) ⊕ *R*_*c*_ ⊕ *T*_*c*_′ ⊕ *h*(*ID*_*i*_||*X*_*s*_) ⊕ *T*_*c*_′ = *R*_*c*_′. If *M*_5_′ = *M*_5_, it ensures that the login request message is a replay one. Otherwise, *S*_*j*_ updates *M*_5_ with *M*_5_′ in its database. Thus, it is noted that the timestamp and random nonces are used together to defend the replay and man-in-the-middle attacks.

*Step A2*. *S*_*j*_ generates a random nonce *R*_*s*_ and then computes *M*_7_ = *M*_4_ ⊕ *R*_*s*_ ⊕ *T*_*s*_, where *T*_*s*_ is the current system timestamp of the server *S*_*j*_, *M*_8_ = *h*(*R*_*s*_||*T*_*s*_||*M*_5_||*T*_*c*_) ⊕ *N* *ID*_*i*_^new^, where *N* *ID*_*i*_^new^ is a random and temporary identity generated by *S*_*j*_, and *M*_9_ = *h*(*ID*_*i*_||*M*_5_ + 1||*T*_*c*_ + 1||*R*_*s*_||*T*_*s*_||*N* *ID*_*i*_^new^). *S*_*j*_ then sends the authentication request message 〈*M*_7_, *M*_8_, *M*_9_, *T*_*s*_〉 to the user *U*_*i*_ via a public channel.

*Step A3*. After receiving the message in Step A2, *C*_*i*_ checks the validity of the timestamp *T*_*s*_ in the received message with the condition |*T*_*s*_ − *T*_s_* | <Δ*T*, where *T*_s_* is the current system timestamp of *C*_*j*_ and Δ*T* the expected transmission delay. If this condition does not hold, the phase terminates immediately. Otherwise, *C*_*i*_ computes

(2)       M10=M7⊕M1⊕Ts      =h(IDi||Xs)⊕Rs⊕Ts⊕h(IDi||Xs)⊕Ts      =Rs,      M11=h(M10||Ts||Rc||Tc),     NIDinew∗=M8⊕M11.

*C*_*i*_ further computes *M*_12_ = *h*(*ID*_*i*_||*R*_*c*_ + 1||*T*_*c*_ + 1||*M*_10_||*T*_*s*_||*N* *ID*_*i*_^new∗^) and checks the condition *M*_12_ = *M*_9_. If it does not hold, this phase terminates immediately. Otherwise, on the other hand, *C*_*i*_ updates *TD*_*i*_ and *D*_*i*_ with *D*_*i*_ and *D*_*i*_ ⊕ *N* *ID*_i_* ⊕ *N* *ID*_*i*_^new∗^, respectively, in its memory.

*Step A4*. *C*_*i*_ computes *M*_13_ = *h*(*M*_10_ + 1||*T*_*s*_ + 1||*R*_*c*_ + 1||*T*_*c*_ + 1||*N* *ID*_*i*_^new∗^||*ID*_*i*_) and sends the authentication acknowledgment message 〈*M*_13_〉 to the server *S*_*j*_ via a public channel. *C*_*i*_ also computes a secret session key shared between *U*_*i*_ and *S*_*j*_ as *SK*_*U*_*i*_,*S*_*j*__ = *h*(*ID*_*i*_||*R*_*c*_||*T*_*c*_||*M*_10_||*T*_*s*_||*M*_1_).

*Step A5*. After receiving the authentication acknowledgment message 〈*M*_13_〉 from the user *U*_*i*_ in Step A4, *S*_*j*_ computes *M*_14_ = *h*(*R*_*s*_ + 1||*T*_*s*_ + 1||*M*_5_ + 1||*T*_*c*_ + 1||*N* *ID*_*i*_^new^||*ID*_*i*_) and verifies whether the condition *M*_14_ = *M*_13_ holds. If it holds, *S*_*j*_ authenticates the user *U*_*i*_ and also computes the same secret session key shared with *U*_*i*_ as *K*_*S*_*j*_,*U*_*i*__ = *h*(*ID*_*i*_||*M*_5_||*T*_*c*_||*R*_*s*_||*T*_*s*_||*M*_4_). Thus, after successful authentication, both *U*_*i*_ and *S*_*j*_ can communicate securely using the established secret session key.

**Case 2**

This case remains almost the same as Case 1 except the following in Step A6.

*Step A6*. *N* *ID*_i_* is obtained by computing *h*(*ID*_i_*||*r*_i_*) ⊕ *TD*_*i*_ instead of *h*(*ID*_i_*||*r*_i_*) ⊕ *D*_*i*_ in Step L3 of the login phase. The smart card *C*_*i*_ of the user *U*_*i*_ in this case only needs to update *D*_*i*_ with *D*_*i*_ ⊕ *N* *ID*_i_* ⊕ *N* *ID*_*i*_^new∗^ without changing *TD*_*i*_ in Step A3.

The summary of the authentication phase is given in Table 3.

#### 3.4.4. Password Change Phase

To enhance security, a user *U*_*i*_ needs to change his/her password. Let *U*_*i*_ want to change his/her password *PW*_*i*_ with a new password *PW*_*i*_^new^. For this phase, the following steps are executed by the smart card *C*_*i*_ of the user *U*_*i*_ without contacting the remote server *S*_*j*_.

*Step P1*. *U*_*i*_ first inserts his/her smart card *C*_*i*_ into a card reader of the specific terminal and then inputs identity *ID*_*i*_ and provides old password *PW*_*i*_^old^.

*Step P2*. *C*_*i*_ then computes masked password *R* *PW*_*i*_^old^ = *h*(*ID*_*i*_||*K*||*PW*_*i*_^old^) using the secret number *K* stored in its memory and *r*_*i*_^old^ = *h*(*ID*_*i*_||*R* *PW*_*i*_^old^). *C*_*i*_ checks if the condition *r*_*i*_^old^ = *r*_*i*_ holds. If it does not hold, the old password verification fails and this phase terminates immediately. Otherwise, *C*_*i*_ asks the user *U*_*i*_ to input his/her chosen strong (high-entropy) password *PW*_*i*_^new^, where *PW*_*i*_^old^ ≠ *PW*_*i*_^new^.

*Step P3*. *C*_*i*_ computes

(3) x=ei⊕riold=h(IDi||Xs)⊕ri⊕riold=h(IDi||Xs), since riold=ri,RPWinew=h(IDi||K||PWinew),rinew=h(IDi||RPWinew),einew=x⊕rinew.

*C*_*i*_ further computes *TD*_*i*_^new^ = *TD*_*i*_ ⊕ *h*(*ID*_*i*_||*r*_*i*_^old^) ⊕ *h*(*ID*_*i*_||*r*_*i*_^new^) = *N* *ID*_*i*_ ⊕ *h*(*ID*_*i*_||*r*_*i*_^new^) and *D*_*i*_^new^ = *TD*_*i*_^new^.

*Step P4*. Finally, *C*_*i*_ updates *r*_*i*_ with *r*_*i*_^new^, *e*_*i*_ with *e*_*i*_^new^, *TD*_*i*_ with *TD*_*i*_^new^, and *D*_*i*_ with *D*_*i*_^new^ in its memory.

Thus, it is clear that our scheme provides efficient password change phase in order to change the password of a user *U*_*i*_ at any time locally and correctly without further contacting the remote server *S*_*j*_.

## 4. Security Analysis of the Proposed Scheme

In this section, we first show the correctness of our proposed scheme. We then provide informal and formal security analysis to show that our scheme is secure against various known attacks.

### 4.1. Correctness

In Theorem 1, we provide the correctness of our scheme.

**Theorem 1**

The proposed scheme always establishes the correct secret session key between the user *U*_*i*_ and the server *S*_*j*_ during the authentication phase after the successful mutual authentication between them.

**Proof**

During the authentication phase of our scheme, in Steps A4 and A5, after the successful mutual authentication the user *U*_*i*_ and the server *S*_*j*_ compute a secret session key between them. Note that, in Step A4, *C*_*i*_ computes the secret session key shared between *U*_*i*_ and *S*_*j*_ as *K*_*U*_*i*_,*S*_*j*__ = *h*(*ID*_*i*_||*R*_*c*_||*T*_*c*_||*M*_10_||*T*_*s*_||*M*_1_), where *M*_10_ = *R*_*s*_ and *M*_1_ = *h*(*ID*_*i*_||*X*_*s*_). Thus, *K*_*U*_*i*_,*S*_*j*__ = *h*(*ID*_*i*_||*R*_*c*_||*T*_*c*_||*R*_*s*_||*T*_*s*_||*h*(*ID*_*i*_||*X*_*s*_)).

On the other side, the server *S*_*j*_ in Step A5 computes the secret session key shared with *U*_*i*_ as *K*_*S*_*j*_,*U*_*i*__ = *h*(*ID*_*i*_||*M*_5_||*T*_*c*_||*R*_*s*_||*T*_*s*_||*M*_4_), where *M*_5_ = *R*_*c*_ and *M*_4_ = *h*(*ID*_*i*_||*X*_*s*_). As a result, *SK*_*S*_*j*_,*U*_*i*__ = *h*(*ID*_*i*_||*R*_*c*_||*T*_*c*_||*R*_*s*_||*T*_*s*_||*h*(*ID*_*i*_||*X*_*s*_)) = *SK*_*U*_*i*_,*S*_*j*__. Hence, the theorem follows.

### 4.2. Informal Security Analysis

In this section, through the informal security analysis we show that our scheme has the ability to defend the various known attacks, which are discussed in the following subsections.

#### 4.2.1. Replay Attack

Suppose an attacker intercepts the login request message 〈*N* *ID*_i_*, *M*_2_, *M*_3_, *T*_*c*_〉 during the login phase, where *M*_2_ = *M*_1_ ⊕ *R*_*c*_ ⊕ *T*_*c*_ = *h*(*ID*_*i*_||*X*_*s*_) ⊕ *R*_*c*_ ⊕ *T*_*c*_ and *M*_3_ = *h*(*ID*_i_*||*R*_*c*_||*T*_*c*_), and starts a new session with the message 〈*N* *ID*_i_*, *M*_2_′, *M*_3_′, *T*_*c*_′〉 = 〈*N* *ID*_i_*, *M*_2_, *M*_3_, *T*_*c*_〉. According to our policy, the server *S*_*j*_ stores the pair (*ID*_*i*_, *M*_5_), where *M*_5_ = *R*_*c*_, in its database. *S*_*j*_ first checks the validity of the timestamp *T*_*c*_′ and if it is valid, it further computes *M*_4_′ = *h*(*ID*_*i*_||*X*_*s*_), using its own secret number *X*_*s*_. After that *S*_*j*_ computes, say, *M*_5_′ = *M*_2_′ ⊕ *M*_4_′ ⊕ *T*_*c*_′ = *h*(*ID*_*i*_||*X*_*s*_) ⊕ *R*_*c*_ ⊕ *T*_*c*_′ ⊕ *h*(*ID*_*i*_||*X*_*s*_) ⊕ *T*_*c*_′ = *R*_*c*_′. If *M*_5_′ = *M*_5_, it ensures that the login request message is a replay one. Since the transmission delay time is short, even if the attacker replays the same login request message during that time, our scheme prevents this as a replay message due to verification of random nonce attached to the message with that in the stored database. As a result, both the timestamp and random nonce together help to defend strongly the replay attack in our scheme.

#### 4.2.2. Man-in-the-Middle Attack

Suppose an attacker intercepts the login request message 〈*N* *ID*_i_*, *M*_2_, *M*_3_, *T*_*c*_〉 during the login phase, where *M*_2_ = *M*_1_ ⊕ *R*_*c*_ ⊕ *T*_*c*_ = *h*(*ID*_*i*_||*X*_*s*_) ⊕ *R*_*c*_ ⊕ *T*_*c*_ and *M*_3_ = *h*(*ID*_i_*||*R*_*c*_||*T*_*c*_). In order to make success in the man-in-the-middle attack, the attacker has to change *M*_2_ and *M*_3_ properly so that the server *S*_*j*_ can authenticate the message successfully. Assume that the attacker uses a timestamp *T*_*c*_′ and wants to change *M*_2_ and *M*_3_ to *M*_2_′ = *M*_2_ ⊕ *T*_*c*_ ⊕ *T*_*c*_′ = *h*(*ID*_*i*_||*X*_*s*_) ⊕ *R*_*c*_ ⊕ *T*_*c*_′ and *M*_3_′ = *h*(*ID*_i_*||*R*_*c*_||*T*_*c*_′), respectively. However, for *M*_3_′ the attacker needs to know both *ID*_i_* and *R*_*c*_ which are unknown to that attacker. As pointed out in [20], the probability of guessing an identity composed of exact *n* characters is approximately 1/2^6*n*^. Thus, to correctly know *ID*_i_* and *R*_*c*_ from *M*_3_, the attacker has to guess both *ID*_i_* and *R*_*c*_ at the same time using *T*_*c*_ and the probability of guessing both *ID*_i_* composed of exact *n* characters and *R*_*c*_ composed of *m* bits (*m* = 160 bits in our scheme) at the same time becomes approximately 1/2^6*n*+*m*^. If *n* = 10, then this probability is approximately 1/2^60+160^ = 1/2^220^, which is very negligible. As a result, the attacker does not have any ability to succeed in this attack and, hence, our scheme is secure against the man-in-the-middle attack.

#### 4.2.3. Impersonation Attack

In this attack, the purpose of an attacker is to impersonate the remote server *S*_*j*_ or a legal user *U*_*i*_ in order to cheat the other party. Suppose an attacker intercepts the login request message 〈*N* *ID*_i_*, *M*_2_, *M*_3_, *T*_*c*_〉 during the login phase and wants to start a new session. In order to start a new session, the attacker has to modify both *M*_2_ and *M*_3_. However, as discussed in Section 4.2.2, to change *M*_3_ the attacker has to guess/know both *ID*_*i*_ and *R*_*c*_, which are unknown to the attacker. Thus, the probability of guessing both *ID*_i_* composed of exact *n* characters and *R*_*c*_ composed of *m* bits (*m* = 160 bits in our scheme) at the same time becomes approximately 1/2^6*n*+*m*^ = 1/2^6*n*+160^, which is also very negligible. Hence, our scheme prevents the impersonation attack.

#### 4.2.4. Stolen Smart Card Attack

In this attack, we assume that the smart card *C*_*i*_ of a legal user *U*_*i*_ is lost or stolen by an attacker. Then the attacker can extract all the secret information (*r*_*i*_, *e*_*i*_, *TD*_*i*_, *D*_*i*_, *K*) from the memory of the stolen or lost smart card *C*_*i*_ of the user *U*_*i*_ using the power analysis attacks [23, 24]. Note that *r*_*i*_ = *h*(*ID*_*i*_||*R* *PW*_*i*_) = *h*(*ID*_*i*_||*h*(*ID*_*i*_||*K*||*PW*_*i*_)) and *e*_*i*_ = *h*(*ID*_*i*_||*X*_*s*_) ⊕ *r*_*i*_. The attacker can derive *h*(*ID*_*i*_||*X*_*s*_) = *e*_*i*_ ⊕ *r*_*i*_. In order to know the secret information *X*_*s*_ of the server *S*_*j*_, the attacker needs to guess both *ID*_*i*_ and *X*_*s*_. The probability of guessing both *ID*_*i*_ composed of exact *n* characters and *X*_*s*_ composed of *m* bits (*m* = 1024 bits in our scheme) at the same time becomes approximately 1/2^6*n*+*m*^ = 1/2^6*n*+1024^, which is very negligible. Again, to derive the password *PW*_*i*_ composed of *l* characters, the attacker needs to also guess *ID*_*i*_ using *K*. Thus, the probability of guessing both *ID*_*i*_ composed of exact *n* characters and *PW*_*i*_ composed of exact *l* characters at the same time becomes approximately 1/2^6*n*+6*l*^, which is also negligible. Hence, our scheme prevents the stolen smart card attack.

#### 4.2.5. Password Guessing Attack

In this attack, we consider both offline and online password guessing attacks. As in Section 4.2.4, we assume that the smart card *C*_*i*_ of a legal user *U*_*i*_ is lost or stolen by an attacker and all the secret information (*r*_*i*_, *e*_*i*_, *TD*_*i*_, *D*_*i*_, *K*) stored in the memory of the smart card *C*_*i*_ is known to the attacker. Still then the attacker can not guess correctly the password *PW*_*i*_ of *U*_*i*_ offline, which is evident from Section 4.2.4.

Suppose the attacker intercepts all the transmitted messages 〈*N* *ID*_i_*, *M*_2_, *M*_3_, *T*_*c*_〉 during the login phase and 〈*M*_7_, *M*_8_, *M*_9_, *T*_*s*_〉 and 〈*M*_13_〉 during the authentication phase. However, none of these messages involves the password *PW*_*i*_ of the user *U*_*i*_. As a result, these messages will not be helpful to the attacker to obtain *PW*_*i*_ of *U*_*i*_ online. Thus, our scheme is secure against both offline and online password guessing attacks.

#### 4.2.6. Denial-of-Service Attack

Note that, in our scheme, the smart card *C*_*i*_ of a legal user *U*_*i*_ stores *TD*_*i*_ and *D*_*i*_ for the previous and the latest random identities, respectively. Thus, the corruption of the message 〈*M*_13_〉 during the authentication phase is not possible by an attacker and, hence, our scheme prevents the denial-of-service attack.

#### 4.2.7. User Anonymity

In our scheme, all the transmitted messages include the identity *ID*_*i*_ of a legal user *U*_*i*_ indirectly and it is protected by the one-way secure hash function *h*(·). Due to the collision-resistant property of *h*(·), it is computationally infeasible for an attacker to derive *ID*_*i*_.

Even if we assume that the smart card *C*_*i*_ of a legal user *U*_*i*_ is lost or stolen by an attacker and all the secret information (*r*_*i*_, *e*_*i*_, *TD*_*i*_, *D*_*i*_, *K*) stored in the memory of the smart card *C*_*i*_ is known to the attacker, from *TD*_*i*_ and *N* *ID*_i_* from the intercepted login request message 〈*N* *ID*_i_*, *M*_2_, *M*_3_, *T*_*c*_〉 the attacker can compute *h*(*ID*_*i*_ | |*r*_*i*_) = *TD*_*i*_ ⊕ *N* *ID*_i_*. Again, *ID*_*i*_ is protected by the one-way secure hash function *h*(·). Due to the collision-resistant property of *h*(·), it is computationally infeasible for an attacker to derive *ID*_*i*_. Hence, our scheme preserves the user anonymity property.

#### 4.2.8. Mutual Authentication

During the authentication phase, after receiving the authentication request message 〈*M*_7_, *M*_8_, *M*_9_, *T*_*s*_〉 from the server *S*_*j*_, the smart card *C*_*i*_ of a legal user *U*_*i*_ computes *M*_12_ = *h*(*ID*_*i*_||*R*_*c*_ + 1||*T*_*c*_ + 1||*M*_10_||*T*_*s*_||*N* *ID*_*i*_^new∗^) and checks the condition *M*_12_ = *M*_9_. If it holds, *U*_*i*_ authenticates the server *S*_*j*_ and then only sends the authentication acknowledgment message 〈*M*_13_〉 to the server *S*_*j*_. After that the server *S*_*j*_ also computes *M*_14_ = *h*(*R*_*s*_ + 1||*T*_*s*_ + 1||*M*_5_ + 1||*T*_*c*_ + 1||*N* *ID*_*i*_^new^||*ID*_*i*_) and verifies whether the condition *M*_14_ = *M*_13_ holds. If it holds, *S*_*j*_ authenticates the user *U*_*i*_. Hence, the mutual authentication is always performed in our scheme.

#### 4.2.9. Session Key Security

After mutual authentication, the smart card *C*_*i*_ of a legal user *U*_*i*_ computes the secret session key shared between *U*_*i*_ and *S*_*j*_ as *K*_*U*_*i*_,*S*_*j*__ = *h*(*ID*_*i*_||*R*_*c*_||*T*_*c*_||*M*_10_||*T*_*s*_||*M*_1_). The server *S*_*j*_ also computes the secret session key shared with the user *U*_*i*_ as *K*_*S*_*j*_,*U*_*i*__ = *h*(*ID*_*i*_||*M*_5_||*T*_*c*_||*R*_*s*_||*T*_*s*_||*M*_4_), where *M*_1_ = *h*(*ID*_*i*_||*X*_*s*_) and *M*_4_ = *h*(*ID*_*i*_||*X*_*s*_). It is also evident from Theorem 1 that *K*_*U*_*i*_,*S*_*j*__ = *SK*_*S*_*j*_,*U*_*i*__. In order to compute the secret key *SK*_*U*_*i*_,*S*_*j*__ from all the transmitted messages during the login and authentication phases, an attacker has to guess/derive correctly *ID*_*i*_ composed of exact *n* characters, *X*_*s*_ of *m* = 1024 bits, and *R*_*c*_ and *R*_*s*_, each composed of 160 bits at the same time, and, thus, the probability of deriving this secret key is approximately 1/2^6*n*+*m*+160+160^ = 1/2^6*n*+1344^, which is very negligible. As a result, our scheme also provides the session key security.

### 4.3. Formal Security Analysis

For the formal security analysis, we follow the formal definition of a one-way hash function *h*(·) given in Definition 2.

**Definition 2 (one-way hash function [25, 26])**

A one-way collision-resistant hash function *h* : {0,1}* → {0,1}^*n*^ is a deterministic function that takes the input as an arbitrar-length binary string *x* ∈ {0,1}* and outputs a binary string *y* = *h*(*x*)∈{0,1}^*n*^ of fixed length *n*. We formalize an adversary *A*'s advantage in finding collision in the following manner:

(4) AdvAHASH(t) =Pr[(x,x′)⟸A:x≠x′h(x)=h(x′)],

where Pr[*E*] denotes the probability of an event *E* and (*x*, *x*′) ⇐ *A* denotes that the pair (*x*, *x*′) is selected randomly by *A*. The adversary *A* is allowed to be probabilistic and the probability in the advantage is computed over the random choices made by the adversary *A* with the execution time *t*. The hash function *h*(·) is called collision resistant, if Adv_*A*_^HASH^(*t*) ≤ *ϵ*, for any sufficiently small *ϵ* > 0.

We then define the following random oracle for our formal security analysis.

- *Reveal*. This random oracle will unconditionally output the input *x* from the corresponding hash value *y* = *h*(*x*).

In Theorems 3 and 4, we show that our scheme is secure against an adversary for deriving the secret number *X*_*s*_ of the server and the password *PW*_*i*_ of a user *U*_*i*_.

**Theorem 3**

Under the assumption that a one-way hash function *h*(·) closely behaves like a random oracle, the proposed scheme is provably secure against an adversary for deriving the secret number *X*_*s*_ of the server *S*_*j*_.

**Proof**

We follow the same proof presented in [14, 27, 28]. In this proof, we construct an adversary *A* such that he/she can derive the secret number *X*_*s*_ of the server *S*_*j*_ correctly. For this purpose, the adversary *A* runs the experiment, *EXP*1_*A*,*REUAS*_^*HASH*^, for our robust and effective smart-card-based remote user authentication scheme, say, REUAS given in Algorithm 1.

We now define the success probability for *EXP*1_*A*,*REUAS*_^*HASH*^ as *cc*1_*A*,*REUAS*_^*HASH*^ = Pr[*EXP*1_*A*,*REUAS*_^*HASH*^ = 1] − 1. Then the advantage of *EXP*1_*A*,*REUAS*_^*HASH*^ becomes Adv1_*A*,*REUAS*_^*HASH*^(*t*_1_, *q*_*R*_) = max⁡_*A*_{*S* *u* *cc*1_*A*,*REUAS*_^*HASH*^}, where the maximum is taken over all *A*'s with the execution time *t*_1_ and the number of queries *q*_*R*_ made to the *Reveal* oracle. We call that our scheme is provably secure against the adversary *A* for deriving the secret number *X*_*s*_ of the server *S*_*j*_, if Adv1_*A*,*REUAS*_^*HASH*^(*t*_1_, *q*_*R*_) ≤ *ϵ*, for any sufficiently small *ϵ* > 0.

Consider the experiment provided in Algorithm 1. According to this experiment, if the adversary *A* has the ability to invert the hash function *h*(·), then only he/she can derive the secret number *X*_*s*_ of the server *S*_*j*_ and win the game. However, according to Definition 2, it is a computationally infeasible (hard) problem for inverting a one-way hash function *h*(·). Since Adv_*A*_^*HASH*^(*t*) ≤ *ϵ*, for any sufficiently small *ϵ* > 0, we have Adv1_*A*_^*HASH*^(*t*_1_, *q*_*R*_) ≤ *ϵ*, as it is dependent on the former. As a result, the adversary *A* does not have any ability to derive the secret number *X*_*s*_ of the server *S*_*j*_.

**Theorem 4**

Under the assumption that a one-way hash function *h*(·) closely behaves like a random oracle, the proposed scheme is provably secure against an adversary for deriving the password *PW*_*i*_ of a user *U*_*i*_, even if the smart card *C*_*i*_ of  *U*_*i*_ is lost or stolen by that adversary.

**Proof**

We need to construct an adversary *A* such that he/she can derive the password *PW*_*i*_ of the user *U*_*i*_ correctly after extracting the information stored in the stolen or lost smart card *C*_*i*_ of *U*_*i*_. For this purpose, the adversary *A* runs the experiment, *EXP*2_*A*,*REUAS*_^*HASH*^, which is provided in Algorithm 2.

Similar to the experiment *EXP*1_*A*,*REUAS*_^*HASH*^ given in Algorithm 1, we also define the success probability for *EXP*2_*A*,*REUAS*_^*HASH*^ as *S* *u* *cc*2_*A*,*REUAS*_^*HASH*^ = Pr[*EXP*2_*A*,*REUAS*_^*HASH*^ = 1] − 1 and the advantage of *EXP*2_*A*,*REUAS*_^*HASH*^ as Adv2_*A*,*REUAS*_^*HASH*^(*t*_2_, *q*_*R*_) = max⁡_*A*_{*S* *u* *cc*2_*A*,*REUAS*_^*HASH*^}, where the maximum is taken over all *A*'s with the execution time *t*_2_ and the number of queries *q*_*R*_ made to the *Reveal* oracle. Our scheme is then provably secure against the adversary *A* for deriving the password *PW*_*i*_ of the user *U*_*i*_, if Adv2_*A*,*REUAS*_^*HASH*^(*t*_2_, *q*_*R*_) ≤ *ϵ*, for any sufficiently small *ϵ* > 0.

Now, consider the experiment provided in Algorithm 2. After extracting all the secret information (*r*_*i*_, *e*_*i*_, *TD*_*i*_, *D*_*i*_, *K*) from the memory of the stolen or lost smart card *C*_*i*_ of the user *U*_*i*_, the adversary *A* can derive the password *PW*_*i*_ of the user *U*_*i*_ and win the game, if he/she has the ability to invert the one-way hash function *h*(·). Since inverting the one-way hash function *h*(·) is computationally infeasible, that is, Adv_*A*_^*HASH*^(*t*) ≤ *ϵ*, for any sufficiently small *ϵ* > 0, we have Adv2_*A*_^*HASH*^(*t*_2_, *q*_*R*_) ≤ *ϵ*, as it is dependent on the former. Hence, our scheme is provably secure against an adversary for deriving the password *PW*_*i*_ of a user *U*_*i*_, even if the smart card *C*_*i*_ of *U*_*i*_ is lost or stolen by that adversary.

## 5. Formal Security Verification Using AVISPA Tool

In this section, through the simulation results for the formal security verification using the widely accepted AVISPA tool [20, 21, 27, 28] we show that our scheme is secure against passive and active attacks.

AVISPA (Automated Validation of Internet Security Protocols and Applications) is considered as a push-button tool for the automated validation of Internet security-sensitive protocols and applications [29]. AVISPA has four different back-ends that implement a variety of state-of-the-art automatic analysis techniques. The back-ends are the On-the-Fly Model-Checker (OFMC), Constraint Logic based Attack Searcher (CL-AtSe), SAT-based Model-Checker (SATMC), and Tree Automata based on Automatic Approximations for the Analysis of Security Protocols (TA4SP). The protocols to be analyzed under the AVISPA tool require specifying them in a language, called HLPSL (High Level Protocols Specification Language), which is a role-oriented language. The specification in HLPSL is first translated into a low-level specification by a translator, which is called the hlpsl2if. hlpsl2if generates a specification in an intermediate format, which is known as the intermediate format (IF). The output format (OF) of AVISPA is generated using one of the four back-ends: OFMC, CL-AtSe, STAMC, and TA4SP. The analysis of the OF is made as follows. The first printed section, called SUMMARY, indicates whether the protocol is safe or unsafe or whether the analysis is inconclusive. DETAILS is the second section, which explains under what condition the protocol is declared safe, what conditions have been used for finding an attack, or finally why the analysis was inconclusive. The remaining sections, called PROTOCOL, GOAL, and BACKEND, represent the name of the protocol, the goal of the analysis, and the name of the back-end used, respectively. Finally, at the end of the analysis, after some possible comments and the statistics, the trace of the attack (if any) is also printed in the usual Alice-Bob format. One can find more details on HLPSL in [29].

### 5.1. Specifying Our Scheme

We have implemented our scheme for the formal security verification for the registration phase, the login phase, and the authentication phase using the HLPSL language. We have two basic roles: one for alice, which represents the participant as the user *U*_*i*_, and another for bob, which represents the remote server *S*_*j*_. The role of the initiator, the user *U*_*i*_, is shown in Algorithm 3. In this role, *U*_*i*_ first receives the start signal, changes its state value from 0 to 1, and then sends the registration request message 〈*ID*_*i*_, *R* *PW*_*i*_〉 securely to the server *S*_*j*_ using the symmetric key *SK* *u* *i* *s* *j* shared between *U*_*i*_ and *S*_*j*_ via the *Snd*(  ) operation. During the registration phase, the user *U*_*i*_ then receives a smart card containing the information {*r*_*i*_, *e*_*i*_, *TD*_*i*_, *D*_*i*_, *h*(·)} securely from *S*_*j*_ by the *Rcv*(  ) operation. The type declaration channel (*dy*) in HLPSL specification declares that the channel is for the Dolev-Yao threat model [1]. In this role,* agent* represents a principal name. The intruder is always assumed to have the special identifier *i*.* symmetric_key* represents a key for a symmetric-key cryptosystem.* text* is often used as nonce. This value can be also used for messages.* nat* type represents the natural numbers in nonmessage contexts, whereas* const* represents a constant.* hash_func* represents cryptographic hash functions. function also represents functions on the space of messages. In HLPSL, it is assumed that the intruder cannot invert hash functions (in essence, that they are one way). The space of legal messages is defined as the closure of the basic types. For example, given a message* Msg* and an encryption key* Key*, {*Msg*}_*Key* denotes the symmetric/public-key encryption. The associative “·” operator is used for concatenation. The “*played_by A*” declaration tells that the agent named in variable *A* will play a specific role. A knowledge declaration (generally in the top-level* Environment* role) is used to specify the intruder's initial knowledge. Immediate reaction transitions have the form *X* = |>*Y*, which relate an event *X* and an action *Y*. This means that whenever we take a transition that is labeled in such a way so as to make the event predicate *X* true, we must immediately (i.e., simultaneously) execute action *Y*. If a variable *V* remains permanently secret, it is expressed by the goal* secrecy_of V*. Thus, if *V* is ever obtained or derived by the intruder, a security violation will result.

During the login phase of our scheme, the user *U*_*i*_ sends the login request message 〈*N* *ID*_i_*, *M*_2_, *M*_3_, *T*_*c*_〉 to the server *S*_*j*_. During the authentication phase, after receiving the authentication request message 〈*M*_7_, *M*_8_, *M*_9_, *T*_*s*_〉 from *S*_*j*_, *U*_*i*_ sends the authentication acknowledgment message 〈*M*_13_〉 to *S*_*j*_. In this role, witness (A, B, id, E) declares for a (weak) authentication property of *A* by *B* on *E* that agent *A* is witness for the information *E*; this goal will be identified by the constant *id* in the goal section [29]. This expresses that the agent named in variable *B* has freshly generated the value *E* for the agent named in variable *A*. The *id* term is a new constant that identifies the message term upon which the goal should be authenticated. On the other hand, request (B, A, id, E) for a strong authentication property of *A* by *B* on *E* declares that agent *B* requests a check of the value *E*; this goal will be identified by the constant *id* in the goal section [29]. This formalizes *A*'s acceptance of the value *E* as having been generated for him/her by the agent named in *B*.

The role of the responder, the server *S*_*j*_, is shown in Algorithm 4. During the registration phase, after receiving the registration request message 〈*ID*_*i*_, *R* *PW*_*i*_〉 securely from the user *U*_*i*_, *S*_*j*_ then issues a smart card and sends it containing the information {*r*_*i*_, *e*_*i*_, *TD*_*i*_, *D*_*i*_, *h*(·)} securely to *U*_*i*_. During the login phase, after receiving the login request message 〈*N* *ID*_i_*, *M*_2_, *M*_3_, *T*_*c*_〉, *S*_*j*_ sends the authentication request message 〈*M*_7_, *M*_8_, *M*_9_, *T*_*s*_〉 to *U*_*i*_ in the authentication phase. Finally, *S*_*j*_ waits for the authentication acknowledgment message 〈*M*_13_〉 from *U*_*i*_.

Finally, in Algorithms 5 and 6, we have specified the roles for the session and the goal and environment of our scheme. In the session segment, all the basic roles, alice and bob, are instanced with concrete arguments. The top-level role (called the environment) is always defined in the specification of HLPSL language, which has the global constants and a composition of one or more sessions, where the intruder may play some roles as legitimate users. The intruder (*i*) participates in the execution of protocol as a concrete session during the simulation. Goals are given in their own section, which generally comes at the end of a HLPSL specification. We have two secrecy goals and four authentication processes in the specification of HLPSL in our scheme.

- secrecy_of subs1: it represents that *X*_*s*_ is kept secret to the server *S*_*j*_ only.
- secrecy_of subs2: it represents that *PW*_*i*_ and *K* are kept secret to the user *U*_*i*_ only.
- authentication_on alice_bob_tc: *U*_*i*_ (the smart card) generates a timestamp *T*_*c*_. When the server *S*_*j*_ receives *T*_*c*_ in the messages from *U*_*i*_, *S*_*j*_ authenticates *U*_*i*_.
- authentication_on alice_bob_rc: *U*_*i*_ (the smart card) generates a random nonce *R*_*c*_, where *R*_*c*_ is only known to the user *U*_*i*_. When the server *S*_*j*_ receives *R*_*c*_ in the messages from *U*_*i*_, *S*_*j*_ authenticates *U*_*i*_.
- authentication_on bob_alice_ts: *S*_*j*_ generates a timestamp *T*_*s*_. When *U*_*i*_ receives *T*_*s*_ in the messages from *S*_*j*_, *U*_*i*_ authenticates *S*_*j*_.
- authentication_on bob_alice_rs: *S*_*j*_ generates a random nonce *R*_*s*_, where *R*_*s*_ is only known to *S*_*j*_. When the user *U*_*i*_ receives *R*_*s*_ in the messages from *S*_*j*_, *U*_*i*_ authenticates *S*_*j*_.

### 5.2. Analysis of Results

The simulation results of our scheme using the AVISPA web tool [30] for the widely accepted OFMC back-end [31] are shown in Table 7. It is evident from the summary of the results under OFMC back-end that our scheme is safe. Thus, our scheme is secure against the passive attacks and the active attacks.

## 6. Performance Comparison with Related Schemes

In this section, we compare the performance of our scheme with the related recently proposed password-based remote user authentication schemes: Lee and Liu [13], Das and Bruhadeshwar [14], Sonwanshi et al. [3], and Jiang et al. [15].

For communication cost comparison, we assume that the identity of a user/server is 160 bits, the random nonce is 160 bits, the timestamp is 32 bits, and the hash value is 160 bits. Since the security of 163-bit ECC (elliptic curve cryptography) is the same as that for 1024-bit RSA cryptosystem, for Lee-Liu's scheme [13], Das-Bruhadeshwar's scheme [14], and Jiang et al.'s scheme [15] we take the elliptic curve over a 163-bit prime field and the modulus in RSA as 1024 bits. Thus, each elliptic curve point addition and that of multiplication take (163 + 163) = 326 bits as these are again a point in the elliptic curve, whereas the ciphertext in RSA is 1024 bits.

In our scheme, during the login phase, the login request message 〈*N* *ID*_i_*, *M*_2_, *M*_3_, *T*_*c*_〉 requires (160 + 160 + 160 + 32) = 512 bits. During the authentication phase of our scheme, the authentication request message 〈*M*_7_, *M*_8_, *M*_9_, *T*_*s*_〉 requires (160 + 160 + 160 + 32) = 512 bits and, finally, the authentication acknowledgment message 〈*M*_13_〉 requires 160 bits. Summing all these, the total communication cost of our scheme during the login and authentication phases becomes (512 + 512 + 160) = 1184 bits. In Table 4, we have compared the communication cost of our scheme with other related recent password-based schemes [3, 13–15] for the login and authentication phases. It is noted that Sonwanshi et al.'s scheme [3] requires less communication cost as compared to our scheme and other schemes. However, Sonwanshi et al.'s scheme [3] is shown to be insecure against offline password guessing attack and stolen smart card attack, and it also suffers to protect strong replay attacks. On the other hand, our scheme requires less communication cost as compared to [13–15].

In Table 5, we have compared the computation cost of our scheme with other schemes [3, 13–15] for all the phases. In our scheme, the registration phase requires only 4 hash computations. We ignore the cost of the bitwise XOR operation as it is negligible. The login and authentication phases require 14 hash computations, whereas the password change phase requires 6 hash computations. Thus, a total of 24 hash computations are required for all the phases in our scheme. It is noted that the time taken for a hash computation is significantly less as compared to that for modular exponentiation in RSA encryption/decryption and elliptic curve point addition/multiplication [32]. Thus, our scheme performs significantly better in terms of computational costs than Lee-Liu's scheme [13], Das-Bruhadeshwar's scheme [14], and Jiang et al.'s scheme [15]. Though Sonwanshi et al.'s scheme [3] requires less computational cost than our scheme, Sonwanshi et al.'s scheme is insecure.

Finally, we have compared the functionality provided by our scheme with those for other schemes [3, 13–15] in Table 6. From this table, it is clear that our scheme performs better than Lee-Liu's scheme [13] and Sonwanshi et al.'s scheme [3]. Further, our scheme is also comparable to Das-Bruhadeshwar's scheme [14] and Jiang et al.'s scheme [15]. However, Lee-Liu's scheme [13] has several security weaknesses as shown in [14], and Das-Bruhadeshwar's scheme [14] and Jiang et al.'s scheme [15] require more communication and computational costs as compared to our scheme. Further, Sonwanshi et al.'s scheme [3] is insecure against different attacks. Thus, our scheme performs better in terms of various functionalities as compared to Sonwanshi et al.'s scheme [3].

## 7. Conclusion

In this paper, we have proposed a new robust and secure three-factor remote user authentication scheme, which uses the user's identity, the user's password, and the smart card. Our scheme avoids the expensive operations like modular exponentiations and ECC point addition/multiplication operations as used in [13–15]. Our scheme uses the efficient bitwise XOR operations and one-way hash computations. Due to this, our scheme requires significantly less communication and computational overheads as compared to those for other existing schemes. Our scheme supports several extra features as compared to other schemes. Further, through the rigorous informal and formal security analysis, we have shown that our scheme is secure against possible known attacks. In addition, we have performed the simulation for the formal security analysis to check whether our scheme is secure against passive and active attacks. The simulation results stated in this paper clearly show that our scheme is secure against passive and active attacks. Our scheme also supports efficiently the password change phase always locally without contacting the remote server and correctly. As a result, high security and low communication and computational costs make our scheme more suitable for practical applications.

## Figures and Tables

**Algorithm 1:**
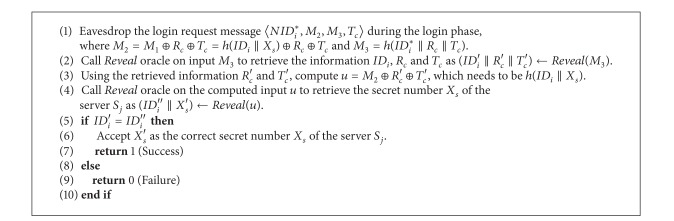
*EX* *P*1_*A*,*REU* *AS*_^*HASH*^.

**Algorithm 2:**
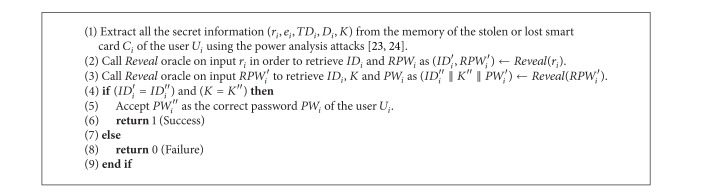
*EX* *P*2_*A*,*REU* *AS*_^*HASH*^.

**Algorithm 3:**
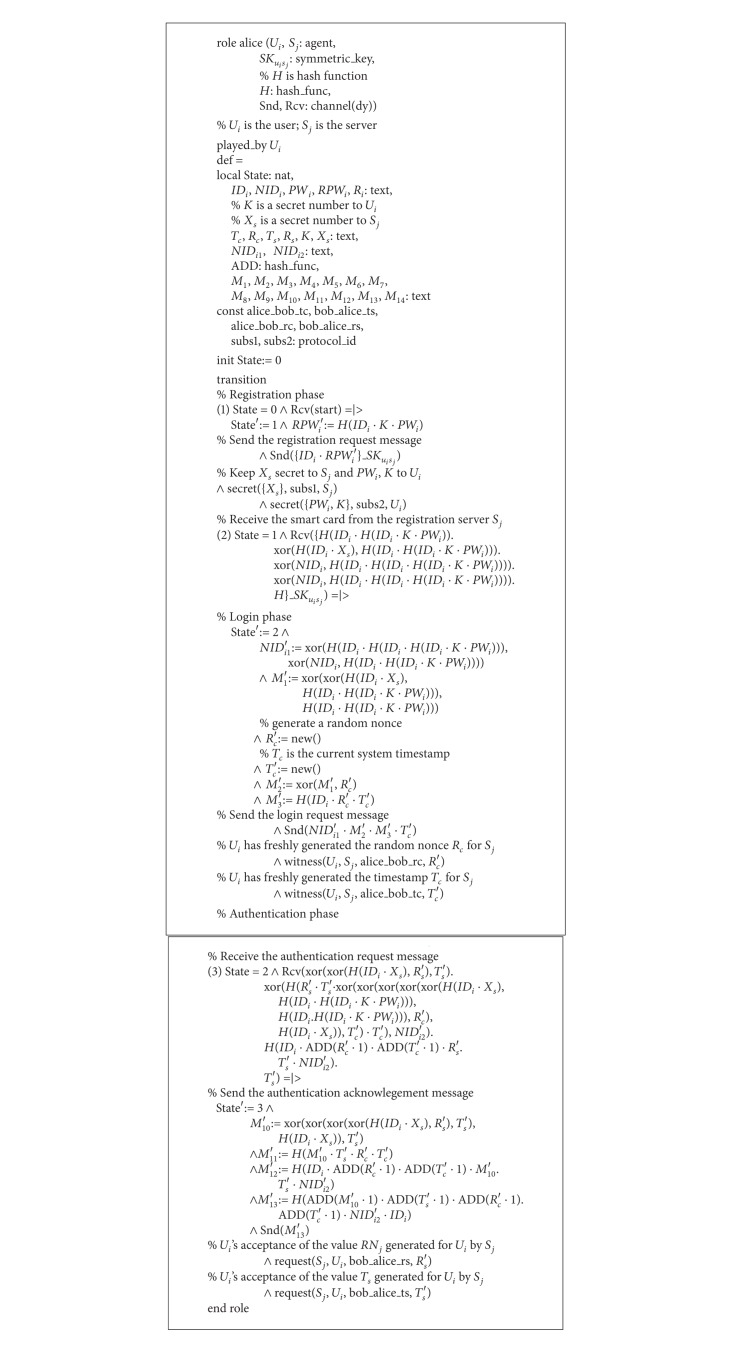
Role specification in HLPSL for the user *U*_*i*_ of our scheme.

**Algorithm 4:**
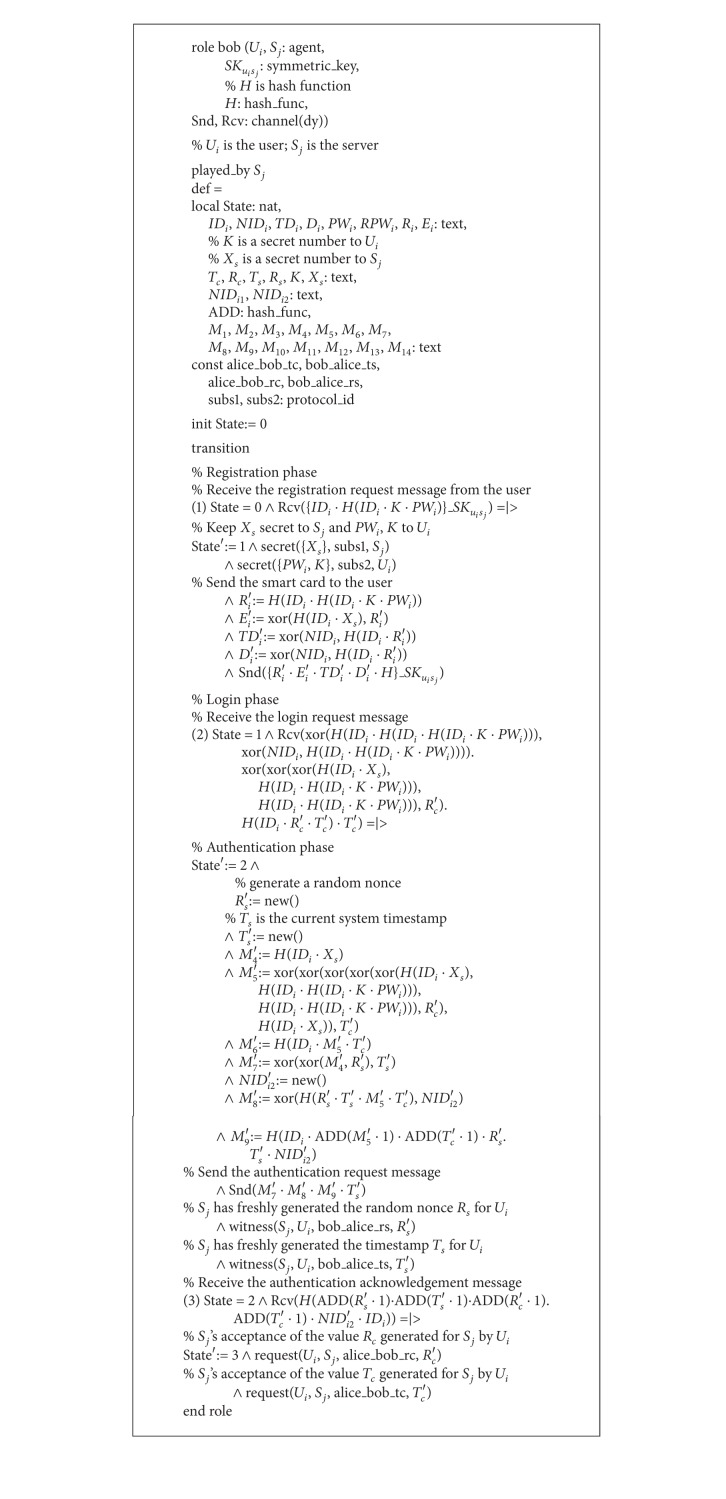
Role specification in HLPSL for the server *S*_*j*_ of our scheme.

**Algorithm 5:**
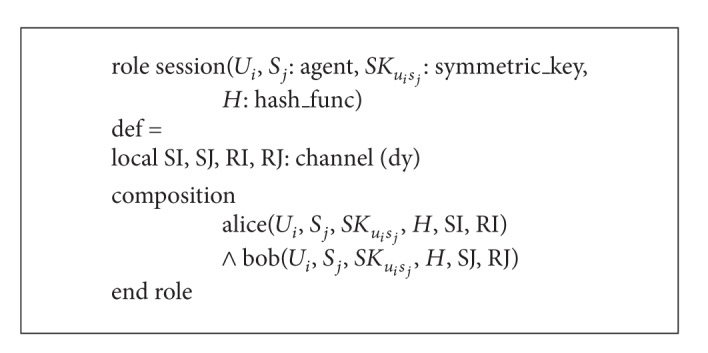
Role specification in HLPSL for the session of our scheme.

**Algorithm 6:**
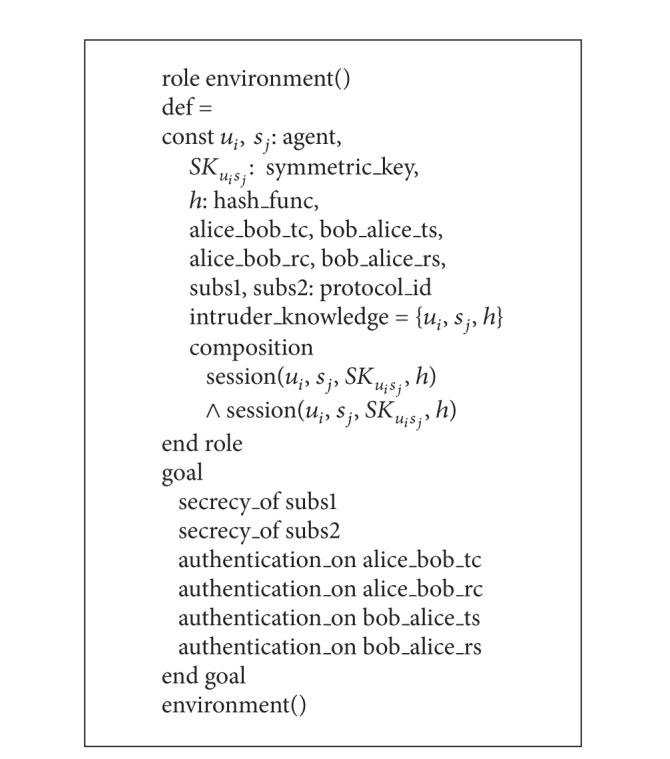
Role specification in HLPSL for the goal and environment of our scheme.

**Table 1:** Summary of the registration phase of our scheme.

User (*U*_*i*_)	Remote server (*S*_*j*_)
Selects *ID*_*i*_, *PW*_*i*_.	
Generates secret number *K*.	
Computes *R* *PW*_*i*_ = *h*(*ID*_*i*_||*K*||*PW*_*i*_).	
$\overset{\langle ID_{i},RPW_{i}\rangle }{\to }$	
(via a secure channel)	Generates secret number *X*_*s*_.
	Computes *r*_*i*_ = *h*(*ID*_*i*_||*R* *PW*_*i*_),
	*e*_*i*_ = *h*(*ID*_*i*_||*X*_*s*_) ⊕ *r*_*i*_,
	*TD*_*i*_ = *N* *ID*_*i*_ ⊕ *h*(*ID*_*i*_||*r*_*i*_), and *D*_*i*_ = *TD*_*i*_.
	$\overset{\;\;\langle SmartCard\left(r_{i},e_{i},TD_{i},D_{i},h\left(\cdot \right)\right)\rangle \;\;}{\leftarrow }$
	(via a secure channel)
Stores *K* into the smart card's memory.	

**Table 2:** Summary of the login phase of our scheme.

User (*U*_*i*_)/smart card (*C*_*i*_)	Remote server (*S*_*j*_)
Inputs *ID*_i_*, *PW*_i_*.	
Computes *R* *PW*_i_* and *r*_i_*.	
Checks if *r*_i_* = *r*_*i*_. If it holds	
computes *N* *ID*_i_* = *h*(*ID*_i_*||*r*_i_*) ⊕ *D*_*i*_,	
*M*_1_ = *e*_*i*_ ⊕ *r*_i_*, *M*_2_ = *M*_1_ ⊕ *R*_*c*_ ⊕ *T*_*c*_,	
and *M*_3_ = *h*(*ID*_i_*||*R*_*c*_||*T*_*c*_).	
$\overset{\;\langle NID_{i}^{*},M_{2},M_{3},T_{c}\rangle \;}{\to }$	
(via a public channel)	

**Table 3:** Summary of the authentication phase of our scheme.

User (*U*_*i*_)/smart card (*C*_*i*_)	Remote server (*S*_*j*_)
	Checks the validity of *T*_*c*_.
	If it holds, computes
	*M*_4_ = *h*(*ID*_*i*_||*X*_*s*_),
	*M*_5_ = *M*_2_ ⊕ *M*_4_ ⊕ *T*_*c*_,
	and *M*_6_ = *h*(*ID*_*i*_||*M*_5_||*T*_*c*_).
	Checks if *M*_6_ = *M*_3_. If it holds,
	computes *M*_7_ = *M*_4_ ⊕ *R*_*s*_ ⊕ *T*_*s*_,
	*M*_8_ = *h*(*R*_*s*_||*T*_*s*_||*M*_5_||*T*_*c*_) ⊕ *N* *ID*_*i*_^new^,
	and *M*_9_ = *h*(*ID*_*i*_||*M*_5_ + 1||*T*_*c*_ + 1
	||*R*_*s*_||*T*_*s*_||*N* *ID*_*i*_^new^).
	$\overset{\;\;\;\langle M_{7},M_{8},M_{9},T_{s}\rangle \;\;\;}{\leftarrow }$
Checks the validity of *T*_*s*_.	(via a public channel)
If it holds, computes	
*M*_10_ = *M*_7_ ⊕ *M*_1_ ⊕ *T*_*s*_,	
*M*_11_ = *h*(*M*_10_||*T*_*s*_||*R*_*c*_||*T*_*c*_),	
*NI* *D*_*i*_^new∗^ = *M*_8_ ⊕ *M*_11_,	
and *M*_12_ = *h*(*ID*_*i*_||*R*_*c*_ + 1||*T*_*c*_ + 1||*M*_10_||*T*_*s*_	
||*N* *ID*_*i*_^new∗^). Checks if *M*_12_ = *M*_9_. If it	
holds, updates *TD*_*i*_ and *D*_*i*_. Computes	
*M*_13_ = *h*(*M*_10_ + 1||*T*_*s*_ + 1||*R*_*c*_ + 1||*T*_*c*_ + 1||*N* *ID*_*i*_^new∗^||*ID*_*i*_).	

$\overset{\langle M_{13}\rangle }{\to }$	
(via a public channel)	Computes *M*_14_ = *h*(*R*_*s*_ + 1||*T*_*s*_ + 1
	||*M*_5_ + 1||*T*_*c*_ + 1||*N* *ID*_*i*_^*new*^||*ID*_*i*_).
	Checks if *M*_14_ = *M*_13_. If it holds,
	*S*_*j*_ authenticates *u*_*i*_.
Computes *K*_*U*_*i*_,*S*_*j*__ = *h*(*ID*_*i*_||*R*_*c*_||*T*_*c*_||*M*_10_||*T*_*s*_||*M*_1_).	Computes *K*_*S*_*j*_,*U*_*i*__ = *h*(*ID*_*i*_||*M*_5_||*T*_*c*_||*SR*_*s*_||*T*_*s*_||*M*_4_).

**Table 4:** Comparison of communication overhead between our scheme and other related schemes during the login and authentication phases.

Scheme	Total number of messages required	Total number of bits required
Lee and Liu [13]	3	1504
Das and Bruhadeshwar [14]	3	1664
Sonwanshi et al. [3]	2	704
Jiang et al. [15]	3	1944
Ours	3	1184

**Table 5:** Comparison of computational overhead between our scheme and other schemes during all phases.

Phase	[13]	[14]	[3]	[15]	Ours
Registration	2*t*_*h*_	4*t*_*h*_	2*t*_*h*_	7*t*_ecm_ + 6*t*_eca_ + 8*t*_*h*_	4*t*_*h*_
Login + authentication	2*t*_me_ + 10*t*_*h*_	2*t*_me_ + 14*t*_*h*_	13*t*_*h*_	10*t*_ecm_ + 3*t*_eca_ + 10*t*_*h*_	14*t*_*h*_
Password change	2*t*_*h*_	5*t*_*h*_	4*t*_*h*_	2*t*_ecm_ + 2*t*_eca_ + 8*t*_*h*_	6*t*_*h*_

Total	2*t*_me_ + 14*t*_*h*_	2*t*_me_ + 23*t*_*h*_	19*t*_*h*_	19*t*_ecm_ + 11*t*_eca_ + 26*t*_*h*_	24*t*_*h*_

Note: *t*_*h*_: the time to compute a one-way hash function; *t*_me_: the time to compute a modular exponentiation; *t*_ecm_: the time to compute a point multiplication on the elliptic curve group; *t*_eca_: the time to compute a point addition on the elliptic curve group.

**Table 6:** Functionality comparison between our scheme and other schemes.

Functionality	[13]	[14]	[3]	[15]	Ours
*F*_1_	No	Yes	No	Yes	Yes
*F*_2_	Yes	Yes	Yes	Yes	Yes
*F*_3_	No	Yes	Yes	Yes	Yes
*F*_4_	Yes	Yes	Yes	Yes	Yes
*F*_5_	Yes	Yes	No	Yes	Yes
*F*_6_	Yes	Yes	No	Yes	Yes
*F*_7_	Yes	Yes	No	No	Yes
*F*_8_	Yes	Yes	No	Yes	Yes
*F*_9_	Yes	Yes	No	Yes	Yes
*F*_10_	Yes	Yes	No	Yes	Yes
*F*_11_	No	Yes	No	No	Yes
*F*_12_	No	Yes	No	No	Yes
*F*_13_	No	Yes	No	Yes	Yes
*F*_14_	No	Yes	Yes	Yes	Yes
*F*_15_	No	Yes	Yes	Yes	Yes
*F*_16_	No	No	No	No	No

Notes: *F*_1_: whether it protects against strong replay attacks or not; *F*_2_: whether it protects against man-in-the-middle attacks or not; *F*_3_: whether it protects against privileged insider attacks or not; *F*_4_: whether it protects against impersonation attacks or not; *F*_5_: whether it protects against stolen smart card attacks or not; *F*_6_: whether it protects against password guessing attacks or not; *F*_7_: whether it protects against denial-of-service attacks or not; *F*_8_: whether it provides mutual authentication or not; *F*_9_: whether it provides user anonymity property or not; *F*_10_: whether it establishes a secret session key between *U*_*i*_ and *S*_*j*_ after successful authentication or not; *F*_11_: whether it provides formal security proof or not; *F*_12_: whether it provides formal security verification or not; *F*_13_: whether it provides session key security or not; *F*_14_: whether it supports local password verification or not; *F*_15_: whether it provides password changing freely and correctly or not; *F*_16_: whether it requires any password verification table or not.

**Table 7:** The result of the analysis using OFMC of our scheme.

% OFMC	
% Version of 2006/02/13	
SUMMARY	
SAFE	
DETAILS	
BOUNDED_NUMBER_OF_SESSIONS	
PROTOCOL	
/home/avispa/web−interface−computation/	
./tempdir/workfiletnHXFr.if	
GOAL	
as_specified	
BACKEND	
OFMC	
COMMENTS	
STATISTICS	
parseTime: 0.00 s	
searchTime: 0.30 s	
visitedNodes: 13 nodes	
depth: 4 plies	
